# Supplementary material for: Predictors and outcomes of cardiac dyssynchrony among patients with heart failure attending Benjamin Mkapa Hospital in Dodoma, central Tanzania: A protocol of prospective-longitudinal study
Source: PLoS One. 2023 Nov 17;18(11):e0287813. doi: 10.1371/journal.pone.0287813 (PMC10655995; doi:10.1371/journal.pone.0287813)
Supplement: Attachment — Submitted filename: RESPONSE TO REVIEWERS.docx [file pone.0287813.s001.docx]

RESPONSE TO REVIEWERS

| **S/N** | **Comment(s)** | **Response** | **Reference Page** |
| --- | --- | --- | --- |
|  | **Editors’ comments** |  |  |
| 1 | Making sure that the manuscript meets the PLOS ONEs requirements | Addressed in whole document |  |
| 2 | To include full ethics statement in methods, including the full name of the IRB or ethics committee who approved or waived your study, as well as whether or not you obtained informed written or verbal consent. | The name of the instructional research review and ethical committee has been added, and the consent type that will be obtained explained | Pg 18;  Line 410-414 |
| 3 | To specify where the minimum data set underlying the results can be found | The availability of data statement has been added in the revised manuscript | Page 18  Line 421-423 |
| 4 | ORCID iD for the corresponding author | ORCiD has been added in revised manuscript | page 1  line 21 |
|  |  |  |  |
|  | **Reviewer 1 comments** |  |  |
| 1 | To change “yrs” to “years” | The word has been rewritten into a full form “years” | Page 2;  Line 32 |
| 2 | Rearranging the paragraph of the rationale “This will offer convincing justification for developing therapeutic services (CRTDs) in our area, incorporating them into local guidelines, and persuading the health insurance companies to include them in their packages” to be the concluding one | The statements/paragraphs have been rearranged and written properly in chronology | Page 4;  Line 88-93 |
|  | To put references for the statement “Despite few studies conducted in Nigeria and Ivory Coast on dyssynchrony in heart failure……” | The reference has been added to the statement | Page 4;  Line 85 |
| 3 | The reviewer wished if the manuscript could be numbered from the first draft | The numbering and track changes has been made available in the following versions |  |
| 5 | To avoid repetition of the words, especially “used” in sample size estimation | The word has been omitted and a statement written correctly | Page 6  Line 123 |
| 6 | Using the appropriate tense in writing the protocol, a future tense to be observed. | “All patients who will be able to provide informed consent, and for those who will be unable, a surrogate relative will consent on their behalf. | Page 7  Line 156 |
| 7 | Reviewer wanted clarification if it is ethically allowed for surrogate relative to consent on behalf for those who will be un able? | In this protocol we abided to “The Mental Capacity Act 2005” provides a legal framework for acting and making decisions on behalf of adults who lack the capacity to make particular decisions for themselves. | Page 7  Line157  Reference number 24 |
| 8 | Reviewer wanted additional information to be added on assessment of electrical dyssynchrony to get a good chronological flow of information | The information has been added on the device operation on assessing electrical dyssynchrony | Page 8  Line 190-191 |
| 9 | The word “closer” to be changed to “closure” in assessment of mechanical dyssynchrony. | The word has been changed | Page 8  Line 201 |
| 10 | Rewriting the sentence in appropriate tense | The sentence was changed to “The time between the start of the QRS complex and the start of the aortic flow velocity detected in the apical five-chamber image will be used to compute the aortic preejection time, termed as LPEI | Page 8  Line 208 |
| 11 | The reviewer suggested showing at which stage data such as BMI (obesity status are obtained (i.e from file by using questionnaire/measurement) and also how BMI will be categorized. | The information has been added on the part of methodology on assessing the predictors of cardiac dyssynchrony | Pg 13,  Line 328-342 |
| 12 | Reviewer suggested the flow of writing to be amended for the Study variables (study aims and summary tables for all aims to come after Data collection process and before Data management, to allow good flow and easiness of reader to follow. | The flow has been amended | Page 14-16  Line 358-380 |
| 13 | The reviewer wanted the information regarding the predictors of cardiac dyssynchrony where and how will be obtained | How and where the information regarding predictors of cardiac dyssynchrony will be obtained has been added to the manuscript. | Pg 12-13, line 391-342 |
| 14 | To specify the detail of the automate BP machine to be used | The specifications have been added on the manuscript | Pg 13, line 347 |
| 15 | To add reference for the statistical software that will be used i.e IBM SPSS PC version 26 | The reference has been added | Pg 17, line 392 |
| 16 | References regarding the information “Interestingly, studies have shown that even though these people have severe heart failure, if they receive cardiac resynchronization therapy (CRT), they do better, have a lower risk of recurrent hospitalization, and lower mortality rate” to be added | The references have been added | Pg19, line 449 |
